# Protocol for the development of joint attention-based subclassification of autism spectrum disorder and validation using multi-modal data

**DOI:** 10.1186/s12888-023-04978-4

**Published:** 2023-08-15

**Authors:** Chanyoung Ko, Soyeon Kang, Soon-Beom Hong, Yu Rang Park

**Affiliations:** 1 Department of Biomedical Systems Informatics, Yonsei University College of Medicine, Seoul, South Korea; 2 Division of Child and Adolescent Psychiatry, Department of Psychiatry, Seoul National University Hospital, Seoul, South Korea; 3 Department of Psychiatry and Institute of Human Behavioral Medicine, Seoul National University College of Medicine, Seoul, South Korea

**Keywords:** Autism spectrum disorder, Multi-modality, Subclassification, Computer vision

## Abstract

**Background:**

Heterogeneity in clinical manifestation and underlying neuro-biological mechanisms are major obstacles to providing personalized interventions for individuals with autism spectrum disorder (ASD). Despite various efforts to unify disparate data modalities and machine learning techniques for subclassification, replicable ASD clusters remain elusive. Our study aims to introduce a novel method, utilizing the objective behavioral biomarker of gaze patterns during joint attention, to subclassify ASD. We will assess whether behavior-based subgrouping yields clinically, genetically, and neurologically distinct ASD groups.

**Methods:**

We propose a study involving 60 individuals with ASD recruited from a specialized psychiatric clinic to perform joint attention tasks. Through the examination of gaze patterns in social contexts, we will conduct a semi-supervised clustering analysis, yielding two primary clusters: good gaze response group and poor gaze response group. Subsequent comparison will occur across these clusters, scrutinizing neuroanatomical structure and connectivity using structural as well as functional brain imaging studies, genetic predisposition through single nucleotide polymorphism data, and assorted socio-demographic and clinical information.

**Conclusions:**

The aim of the study is to investigate the discriminative properties and the validity of the joint attention-based subclassification of ASD using multi-modality data.

**Trial registration:**

Clinical trial, KCT0008530, Registered 16 June 2023, https://cris.nih.go.kr/cris/index/index.do.

**Supplementary Information:**

The online version contains supplementary material available at 10.1186/s12888-023-04978-4.

## Background

The early onset [1], diverse clinical and neurobiological features [2–4], and rising prevalence of autism spectrum disorder (ASD) [5, 6] highlight the urgent need and associated challenges for its subclassification. Recent advancements in genomics [7–9] and neurobiology [10–13] underscore the disorder’s complexity, revealing various etiological pathways. This wide spectrum of ASD symptoms poses both a hurdle and incentive for subclassification. Subclassification methodologies are distinguished by the type of data analyzed and the algorithms used for grouping [12]. The data can be behavior-based or biology-based, each indirectly indicating variations in the other [14, 15]. Moreover, subclassification algorithms range from supervised and unsupervised to hybrid models, each using univariate or multivariate statistics, with their respective pros and cons [16].

### Genomic subclassification of ASD

Genetic factors are the primary risk contributors to ASD, encompassing a range of both rare and common genetic variants. This includes rare de novo mutations, copy number variants, protein-truncating single nucleotide polymorphisms (SNPs), indels, and even non-coding de novo mutations in chromatin interactions [17, 18]. Previous studies have linked these genetic elements to the wide-ranging clinical manifestations of ASD. The genetic origins of ASD are complex and multifactorial; however, our grasp of how these genetic elements functionally influence ASD is still limited. Additionally, the way these genetic factors interact with each other and non-genetic factors to shape risk remains uncertain. However, considering the enormous number of variants implicated in ASD, coupling these genetic data with other types of data obtained through different modalities may help us discern specific genetic vulnerabilities and their corresponding functional consequences in ASD [19].

### Neuro-subclassification of ASD

A few studies have explored neuroanatomical differences between individuals with ASD and neurotypical individuals, as well as within the ASD group itself [11–13, 20]. A significant portion of this research has been conducted using the Autism Brain Imaging Data Exchange (ABIDE) dataset, an open-source compilation of brain imaging data from ASD and neurotypical individuals [21, 22]. Using this resource, Hong et al. identified three distinct ASD subclasses with varying clinical outcomes, based on cortical thickness, intensity contrast, surface area, and geodesic distance [20]. Choi et al., applying an unsupervised clustering approach to ABIDE’s resting state fMRI (rsfMRI) data and utilizing ‘connectome-based gradient’ and ‘functional random forest’ methodologies, were able to differentiate various forms of ASD [12]. However, their results lacked complete replicability as the clinical scores of the subgroups identified through cluster analysis in a follow-up dataset deviated from those in the original dataset. Additionally, these ASD clusters failed to show significant differences in neural connectivity [12, 20]. These outcomes imply that purely unsupervised clustering using intricate data like fMRI might not yield clinically meaningful ASD subtypes.

### Clinical score-driven subclassification of ASD

A research team adopted a top-down approach in their pursuit of developing a new ASD subclassification method, using a clinical-score based clustering method and multimodality data [23]. While this approach allowed for the identification of distinct ASD subgroups based on balance between social-communicative and restricted repetitive behaviors – the two main symptom domains of ASD), these ASD subgroups showed no evident differences in neural circuitry and genetic components [23].

### Limitations of previous studies

Previous attempts at cluster analysis using unsupervised learning on intricate data such as fMRI did not yield consistent or replicable subclassifications; even with identical experimental parameters, the replication dataset did not reproduce the same ASD subgroups as the original discovery dataset [11–13]. Without any labels to guide the clustering, individuals were grouped into distinct subclusters based on non-linear data patterns that lacked clinical significance. Meanwhile, studies using supervised learning methods for cluster analysis also fell short, failing to identify subgroups with unique clinical phenotypes and neurobiological mechanisms [23]. It is worth noting that these studies used human-rated autism-related scale sub-scores as labels for supervised learning. Although these scores are clinically valid and frequently used, they may not adequately capture objective and qualitative differences in ASD phenotypes.

### Possible objective behavioral biomarker for subclassification of ASD

Joint attention, as indicated by prior research, may serve as an objective behavioral biomarker for ASD subclassification [24, 25]. This term refers to the sharing of attentional focus with others on objects or events and is believed to aid social learning [26, 27]. We have established a digitized method to assess joint attention, implementing a novel protocol to prompt three types of joint attention - initiation of joint attention (IJA), low-level and high-level response to joint attention (RJA_low_ and RJA_high_) - as defined in the Early Social Communication Scales (ESCS) [28]. This method uses video-recorded task-related behaviors [26, 27], which are then utilized to train a deep learning model to identify ASD and evaluate its symptom severity [25].

Identifying the most affected level—biological, neural, or behavioral—in an individual with Autism Spectrum Disorder (ASD) to determine the suitable targeted intervention is a significant challenge. To address this, we have initiated the development of the Yonsei-Seoul Multi-modal Subclassification (YSMS) protocol, which was designed to streamline data collection from newly diagnosed ASD patients. Specifically, the focus is on the patterns of joint attention gaze demonstrated during behavioral tasks, with the aim to discern whether these patterns can differentiate various ASD subtypes, each unique in their biological and neurological attributes. We intend to implement a pilot version of this protocol in an exploratory study involving 60 individuals diagnosed with ASD from June 2023 to May 2024 to compare objective behavioral biomarker-based ASD subgroups using multimodality data. Such endeavor is expected to drive the creation of a novel ASD subclassification system, considering each individual’s biological, neural, and behavioral characteristics.

## Methods

### Recruitment

**Fig. 1:**
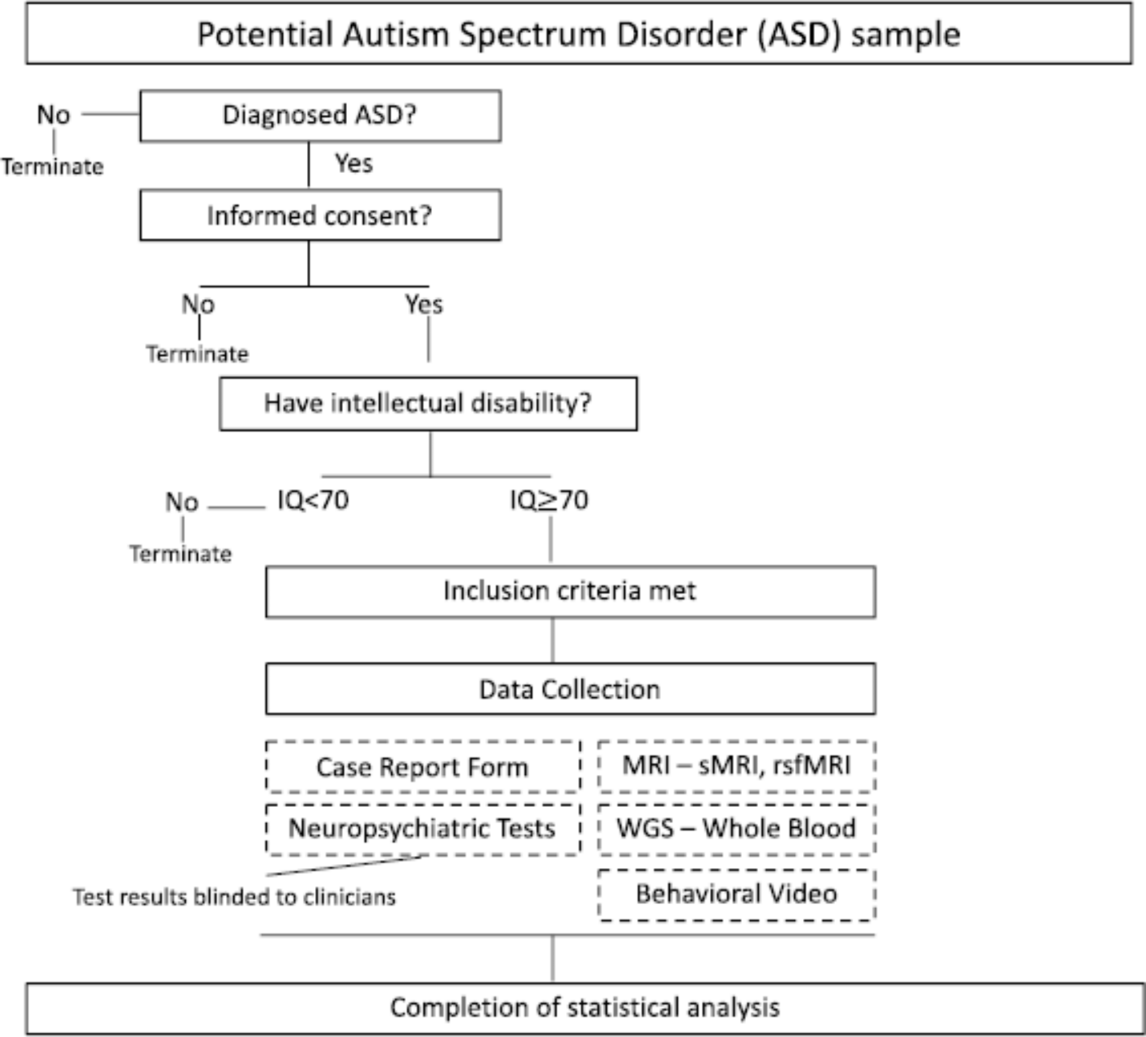
Recruitment and Overall Study Schema

We have designed a single center, prospective, non-randomized experimental study, where individuals with ASD diagnosis will be recruited from June 2023 – May 2024. A group of 60 ASD individuals will be recruited from outpatient clinic at the Child and Adolescent Psychiatry Division of Seoul National University Hospital (SNUH). Enrollment criteria are: (1) age of 30 ~ 71 months, (2) children diagnosed with ASD by a psychiatrist and confirmed by Autism Diagnostic Observation Schedule II (ADOS2). Exclusion criteria are: (1) history of organic brain injury, (2) having visual or auditory deficit, (3) having neurological (motor/musculoskeletal) conditions, (4) history of adverse reaction to sedation anesthesia. A schematic showing the flow of patients is depicted in Fig. 1.

### Multi-modal Data Collection – different types of data

#### Socio-demographics and family history

We gather information about each participant’s socio-demographics and family history to consider for environmental risk factors that may have contributed to different social gaze patterns during joint attention tasks. Patient information such as sex and age at time of assessment will be gathered. Family information such as parental age, parental education level as well as socio-economic status will be gathered from parent interviews. Moreover, number of siblings as well as neuropsychiatric history of siblings will also be obtained.

#### Clinical assessments - neuropsychiatric tests

The diagnosis of ASD is confirmed using ADOS-2, the gold standard diagnostic tool for ASD diagnosis [29]. Autistic tendencies are measured using the Korean versions of the Social Responsiveness Scale (K-SRS) as well as Social Communication Questionnaire (K-SCQ) [30]. The Social Responsiveness Scale (SRS) is a 65-item questionnaire, scored from zero to three based on the frequency of the described behavior, that evaluates the social interactions of children over the past six months, with higher scores indicating lower social capability [30]. The SCQ is a 40-item screening instrument that is based on Autism Diagnostic Interview-Revised (ADI-R), a tool for more in-depth assessment of ASD symptoms, and selects key items that deviate from normal development [30, 31].

Child behavioral problems will be checked with Child Behavior Checklist (CBCL) as well as Vineland Adaptive Behavior Scale (VABS) [32]. The child’s motor functions will be assessed through Developmental Coordination Disorder Questionnaire (DCDQ) [32]. To assess the cognitive levels of participants, the Korean Wechsler Preschool and Primary Scale of Intelligence–Fourth Edition (K-WPPSI-IV) will be used [33].

#### Joint attention tasks and Video Data

We will conduct three types of joint attention tasks in the order of initiation of joint attention, low-level response to joint attention, and high-level response to joint attention according to the Yonsei Seoul Multi-modal Subclassification (YSMS) Joint Attention Task Standard Operating Procedure. Development and validation of AI model trained on video data collected using this protocol has been published elsewhere [24, 25]. Supplementary Fig. 1 shows setup for joint attention experiments based on our proposed protocol. Initiation of joint attention task requires use of only toy 1, while low level response to joint attention task requiring use of toy 1 and toy 2 (distraction) and high-level response to joint attention task requiring use of four picture prompts.

#### Video (RGB or RGB-D)-based Gaze Estimation during Joint attention situations

Following the YSMS Social Gaze-Based Clustering Standard Operating Procedure, we will gather joint attention videoclips for purpose of determining gaze patterns of each participant during joint attention tasks. Joint attention tasks were slightly modified from previous study [24, 25], whereby picture prompts are placed on the same plane as the examiner and toy objects so that all gaze points will fall on the same imaginary vertical hyperplane parallel to the participant’s head/eyes, as shown in Supplementary Fig. 8. With such videorecording set-up, RGB-D information (if using RGB-D device) as well as head’s yaw, pitch, and roll may be computed via computer vision techniques such as algorithm for estimating 3D position of the head and face-tracker [34]. Head position and orientation is tracked through the frame sequence and at every time point this information is stored for later analyzing different patterns of social (joint attention situations) gaze among participants.

#### Magnetic resonance imaging Acquisition and Processing

We use magnetic resonance imaging (MRI) to study differences in brain structure [35], function, and connectivity [36–38]. These methods are important for determining whether different clusters or subgroups of ASD based on social gaze patterns differ at systems-level, providing the underlying neurological mechanisms of different types of ASD. We will be gathering all MRI data from a single center—SNUH.

#### Structural MRI (sMRI) and Diffusion Tensor Imaging (DTI)

MRI scans are acquired on 3T scanners from Siemens (https://www.siemens-healthineers.com/). We will carry out several procedures to optimize structural and functional sequences for the best Siemens-specific options and to address challenges related to standardization and quality assurance of image-acquisition.

Global descriptors of brain anatomy can be elucidated through measures of total grey and white matter volume [35]. Alongside these metrics, we will probe into distinctions in cortical thickness, intensity contrast, geodesic distance, and cortical surface areas. These anatomical indices possess unique genetic determinants, phylogeny, and developmental trajectories [35, 36]. Furthermore, we will calculate structural connectivity indices utilizing both structural MRI scans and Diffusion Tensor Imaging (DTI). Variations in intrinsic grey matter connectivity can be inferred by examining the disparities in both local and global wiring costs [37]. Concurrently, distinctions in short and long-range white matter tracts can be evaluated using tractography analysis of particular pathways [38].

### Resting state functional magnetic resonance imaging (rsfMRI)

Standard gradient-echo echo planar imaging paradigm will be utilized for the acquisition of resting state fMRI scans [39]. We will carry out several procedures to find optimal parameters such as field of view, repetition time, echo time, flip angle, etc. During the resting state scan, participants will be sedated with their eyes closed. The Configurable Pipeline for the Analysis of Connectomes (CPAC, http://fcp-indi.github.com) preprocessing pipeline will be employed for fMRI data processing to remove non-neural noise, such as motion artifacts, from the BOLD-signal [12, 20]. Multiple functional networks have been identified that are characterized by coherent patterns of intrinsic activity between ‘nodes’ that resemble patterns of activity that are engaged during specific cognitive functions [12]. We aim to identify whether social gaze-based subgroups differ in (hyper/hypo-) connectivity within and across these networks [10, 11, 13].

#### Whole genome sequencing (WGS)

We acquire blood samples from the participant for genomic analyses—we specifically wish to identify all variants associated with ASD and calculate polygenic risk scores for each participant. These methods are important for determining whether different clusters or subgroups of ASD based on social gaze patterns differ at the genetic, blueprint-level; ASD individuals may show varying social gaze patterns based on their entire mutation profile.

Collection and Processing of Peripheral Whole Blood Samples: Each participant will contribute a 3ml sample of peripheral whole blood. These samples will be subjected to genomic DNA extraction and whole genome sequencing, performed with the NextSeq 550Dx System (Illumina, San Diego, CA, USA). The procedure for whole genome sequencing will adhere to an industry standard quality-controlled sequence analysis pipeline and utilize a current reference sequence for the purposes of mapping and variant calling [40].

Identification of single nucleotide polymorphisms (SNPs) and insertions and deletions (indels) will be conducted with HaplotypeCaller and MuTect2 from the GATK package (3.8-0, https://github.com/broadinstitute/gatk/releases), as well as with VarScan2 (2.4.0, https://github.com/dkoboldt/varscan/releases). A variety of databases will be used for the analysis and annotation of variants, including but not limited to, the Online Mendelian Inheritance in Man (OMIM), the Human Gene Mutation Database (HGMD), Clinvar, dbSNP, 1000 Genomes, the Exome Aggregation Consortium (ExAC), the Exome Sequencing Project (ESP), the Korean Reference Genome Database (KRGDB), Autism Speaks MSSNG resource, and the Simons Simplex Collection (SSC). Variants will be classified following the standards and guidelines set forth by the American College of Medical Genetics (ACMG) [41]. Following annotation, the sequencing data will undergo additional processing to compute polygenic risk scores for ASD of the top SNPs after thresholding the SNP component [42]. Such computation aims to aid in differentiating between individuals with Autism Spectrum Disorder (ASD) based on their distinct genetic susceptibility or polygenic risk variability.

The different types of data for comparison between subgroups of ASD are listed in Table 1.

**Table 1:** Different types of data acquired in proposed study

Data Types	Case Report Form	Neuro-psychiatric Tests	Brain Imaging	Whole Genome Sequencing	Video
Contents	- Age/sex - Parental age - Parental socio-economic status & education - Number of siblings - ASD in siblings - Diagnosis - Comorbidities	- WPPSI - ADOS2 - SRS - SCQ - CBCL - VABS - DCDQ	- SIEMENS Magnetom Trio 3.0T - sMRI - DTI - rsfMRI - DICOM file	- Source: whole blood - GATK - Variant call format file (annotation)	- RGB or RGB-D camera - Joint attention tasks for ~ 5 min - RGB (color), depth files

## Considerations related to the statistical analysis

### Statistical models and analyses

#### Social Gaze-based semi-supervised clustering analysis

From our previous study, we explored certain relationships between individuals with ASD and clinical scores as well as their performance on joint attention tasks, namely initiation of joint attention and response to joint attention [24, 25]. Such analyses revealed that variables that explain for the variance within group of ASD individuals is their performance in joint attention tasks. Hence forth, we will use join attention success rate for the three different joint attention tasks and perform principal component analysis followed by semi-supervised k-means clustering analysis [43] - using good and poor gaze response head-pose values as labels - to obtain two ASD subgroups, one with decreased joint attention and the other with intact joint attention. An alternative method would be to use a range of threshold value *k* to divide the group of ASD individuals in two sub-groups where one group shows relatively better joint attention abilities.

#### Comparison between good gaze response group and poor gaze response group

Concerning group comparison between two subgroups of ASD based on gaze response during joint attention tasks, several elements will be investigated. These include socio-demographic and clinical characteristics, social gaze patterns (gaze frequency towards or away from foci of attention). Furthermore, structural MRI profiles comprising of cortical thickness and surface area, intensity contrast, and geodesic distance, as well as rsfMRI profiles detailing stepwise functional connectivity difference, will be assessed.

Continuous variables will be represented using means, standard deviations, medians, and ranges. To compare categorical variables, the chi-squared test will be utilized. All statistical analyses and computations of the validation measures will be conducted using Python 3.6.8, in conjunction with SciPy version 1.4.1 (https://docs.scipy.org/doc/scipy-1.4.1/) and Statsmodels 0.11.1 (https://www.statsmodels.org/dev/release/version0.11.1.html). A p-value < 0.05 will be used as the threshold for statistical significance.

#### Power calculation – sample size estimation

For this exploratory study, the lack of preceding data precludes the establishment of a definitive sample size. Nevertheless, referencing guidelines for comparable studies, we advocate a minimum sample size of 12, considering the study’s feasibility, precision of means and variances, and compliance with regulatory requirements [44]. Reflecting on a previous investigation that effectively classified three distinct severity subclasses of ASD with a cohort of 45 individuals [25], our study plans to recruit 60 participants. This number takes into consideration potential participant dropouts or missing data essential for statistical analysis.

### Ethical considerations

This study, adhering to the Helsinki Declaration and its subsequent amendments, mandates informed consent from participants and their legal guardians based on verbal and written details provided. Participants and their legal guardians reserve the right to withdraw at any point, with no obligation to justify their decision and no impact on future treatment. Approval for the study has been granted by the Seoul National University Hospital IRB Review Board (IRB No. H-2210-137-1374). The investigators anticipate no discomfort for the subjects from the tests and tasks involved, with no short- or long-term risks identified in relation to this study.

### Outcome

We posit that ASD individuals with decreased joint attention, characterized by atypical social gaze patterns, will exhibit unique genetic, structural, and functional neural patterns distinct from ASD individuals with mostly intact joint attention, who demonstrate appropriate social gaze patterns. The differentiation is likely rooted in the connectivity within the visual and social brain pathways. It is anticipated that ASD individuals with decreased joint attention will possess SNPs associated with the visual pathway. Furthermore, individuals with greater RRB than SC impairment are expected to exhibit less impairment in social gaze ability. Conversely, participants with a higher SC impairment relative to RRB are predicted to demonstrate a pronounced deficiency in social gaze ability. A layout of expected outcome table is depicted in Table 2.

**Table 2:** Expected outcome table layout

ASD with decreased joint attention	ASD with intact joint attention
**Genetic profile**: SNP component, Unique genes	
**sMRI/DTI**: Cortical features, Volume, Structural connectivity indices	
**rsfMRI**: Connectome gradient, Functional connectivity	
**Clinical/SES profile**: FSIQ, VIQ, SRS, SCQ, CBCL, VABS, DCDQ Parent, Sibling info	
**Social gaze pattern**: Gaze frequency (% Object of interest, % Human, %Other)	

## Discussion

Prompted by a clinical demand for ASD subclassification based on distinct neurobiological and behavioral traits, this study is the first to prospectively gather multi-modal data of ASD individuals, utilizing objective behavioral biomarkers for group clustering. Greater comprehension of ASD types, characterized by their unique behavioral anomalies and underlying mechanisms, is essential for devising patient-specific treatments. Ignoring the interplay of patient-specific needs, overt impairments (social-communication or attentiveness to social cues), genetic susceptibilities, and biological-neurological conditions can result in inefficiencies and parental frustration. Our methodology seeks to mitigate these issues by facilitating successful ASD subclassification based on unique behavioral-neurological-genetic patterns.

### Electronic supplementary material

Below is the link to the electronic supplementary material.

Supplementary Material 1. Joint attention task standard operating procedure.

Supplementary Material 2. Social gaze-based clustering analysis standard operating procedure.

## Data Availability

The dataset used and/or analyzed during the current are available from the corresponding author on reasonable request.

## Abbreviations

ADOS2: Autism Diagnostic Observation Schedule-2
ASD: Autism Spectrum Disorder
CBCL: Child Behavior Checklist
DCDQ: Developmental Coordination Disorder Questionnaire
DTI: Diffusion tensor imaging
FSIQ: Full Scale Intelligence Quotient
GATK: Genome Analysis Toolkit
IJA: Initiation of Joint Attention
JA: Joint Attention
PCA: Principal Component Analysis
RJA_low_: Low Level Response to Joint Attention
RJA_high_: High Level Response to Joint Attention
rsfMRI: Resting State Functional Magnetic Resonance Imaging
SCQ: Social Communication Questionnaire
SNP: Single Nucleotide Polymorphism
SRS: Social Responsiveness Scale
sMRI: Structural Magnetic Resonance Imaging
VABS: Vineland Adaptive Behavior Scales
VIQ: Verbal Intelligence Quotient
WPPSI: Wechsler Preschool and Primary Scale of Intelligence
YSMS: Yonsei Seoul Multi-modal Subclassification
