# Supplementary material for: Protocol for the development of joint attention-based subclassification of autism spectrum disorder and validation using multi-modal data
Source: BMC Psychiatry. 2023 Aug 15;23:589. doi: 10.1186/s12888-023-04978-4 (PMC10426216; doi:10.1186/s12888-023-04978-4)
Supplement: Supplementary file 1 — Supplementary Material 1. Joint attention task standard operating procedure. [file 12888_2023_4978_MOESM1_ESM.docx]

**Joint Attention Task Standard Operating Procedure**

Yonsei Seoul Multi-modal Subclassification (YSMS)

**Contents:**

**Settings**

- **Camera setup for video recording joint attention tasks**
- **Camera setup-related measurements**
- **Video data quality control**
- **Props and equipment**

**Guideline for Eliciting Three Types of Joint Attention**

- **Operational definitions of three types of joint attention**
- **Number of trials per task type**
- **Specific guideline for eliciting each joint attention type**

**Scoring Guideline for Three Types of Joint Attention**

- **Yonsei Joint Attention Scoring Sheet**

**Settings**

**Camera Setup for Video Recording Joint Attention Tasks**

- Use the same camera position and device settings for every behavior task and video recording session.
- Camera-to-participant and examiner-to-participant distances must be fixed and matched for all behavioral tasks and video recording sessions.
- The camera should be placed completely parallel to and directly across from the participant (front facing view with no angle adjustment). The camera should be laid flat on the table, perpendicular to the ground.

**Camera Setup-related Measurements**

- Camera: distance from the edge of table where the participant is seated to camera lens is (__) cm.
- Toy 1: distance from the edge of table where the participant is seated to Toy 1 is (__) cm.
- Toy 2: distance from Toy 1 to Toy 2 is (__) cm.
- Picture 1, 3: distance from the camera to the left wall is (__) cm.
- Picture 2, 4: distance from the camera to the right wall is (__) cm.
- Distance from the ground to the top edge of the bottom two pictures (Pictures 1 & 2) are which is (__) cm.
- Distance from the ground to the top edge of the top two pictures (Pictures 3 & 4) are which is (__) cm.


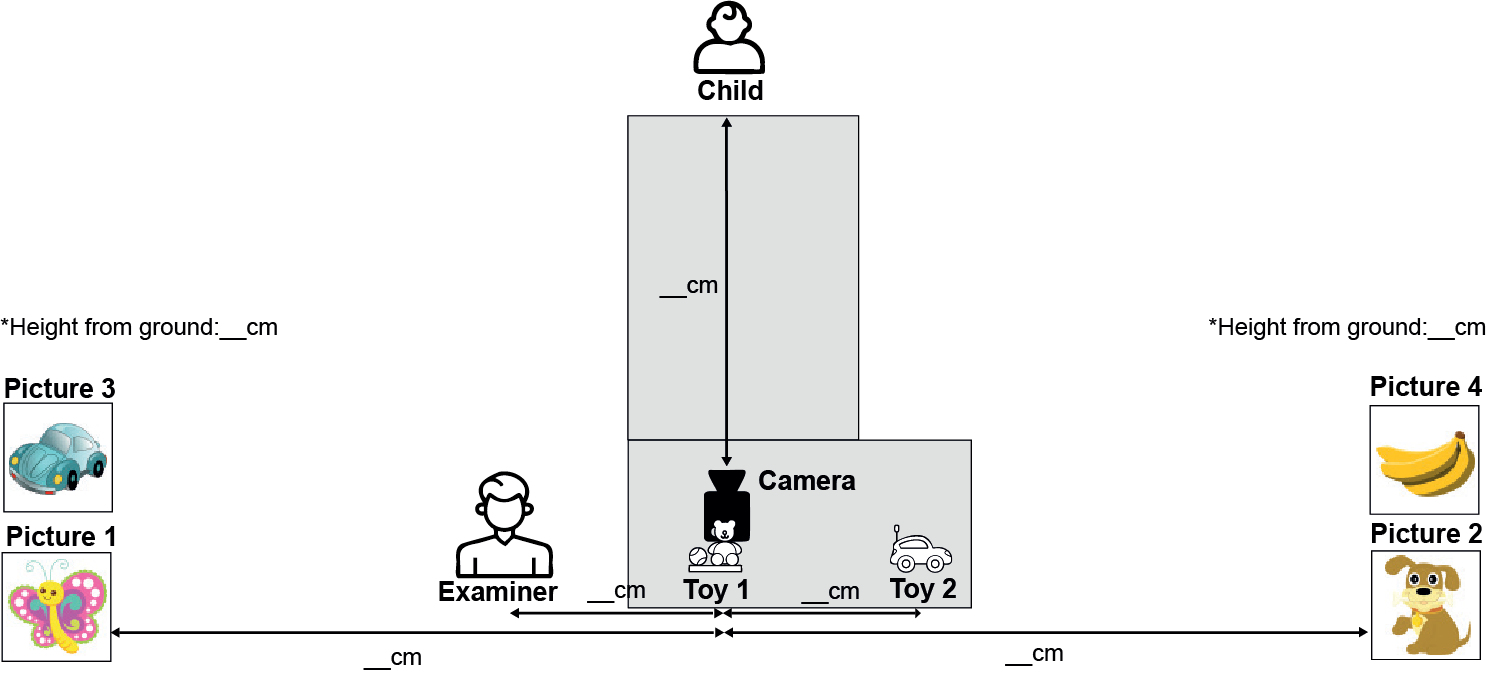


Supplementary figure 1. Camera set-up for video recording joint attention tasks

**Video Data Quality Control—Tips for the Examiner**

- The toy object must be placed as far away from the participant at a distance where the participant can clearly see but cannot simply grab it; joint attention is better elicited in such condition, prompting interaction with the examiner and gaze shifting from the examiner to toy object (or vice versa).
- The examiner’s body parts (hair, head, arm) and toy objects must not obstruct the camera’s view of the participant while conducting the tasks and video recording.
- During response to joint attention (RJA) tasks, examiner must be careful not to obstruct the camera view with their arm when pointing at objects while also making sure their index finger is clearly pointing in the direction of the object (toy or picture) using a pistol grip.
- When pointing to a picture far away (for both the participant and the examiner), examiner may need to extend their arm; while when pointing to Toy 1, the examiner may need to keep their arm close to their body to avoid blocking the camera view.
- The examiner’s finger pointing action itself is a stimulus and should be maintained for at least a full 5 seconds, to give the participant enough time to take notice and respond by turning their head to face and view the object being pointed to.

**Props and Equipment**

- Video recording device i.e. smartphone, digital camera, RGB-D camera

Settings > Camera > Record Video > 1080p HD at 30 fps

- 5 pairs of toy objects of similar sizes and types i.e. 3cm x 5cm x 3cm, dinosaurs or automobiles
- 4 pictures (posters) i.e. A4 size
- Desk, cushion, or chair


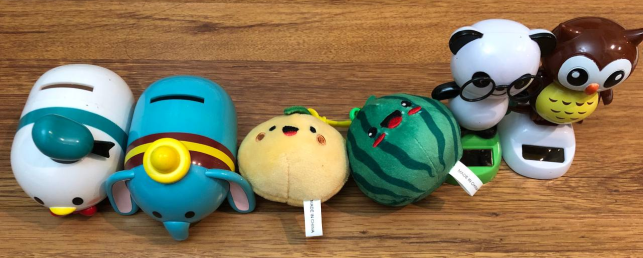


Supplementary figure 2. Examples of toy objects acceptable for initiation of joint attention (IJA) and low level response to joint attention (RJA_low_) tasks

- Examples of picture prompts for high level response to joint attention (RJA_high_) tasks:


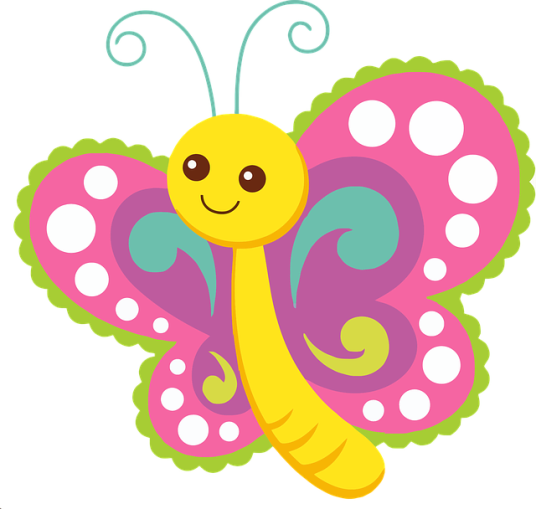


Supplementary figure 3. Picture prompt #1

<https://pixabay.com/ko/illustrations/%EC%95%84%EB%A6%84%EB%8B%A4%EC%9A%B4-%EB%82%98%EB%B9%84-%EA%B7%80%EC%97%AC%EC%9A%B4-%EB%82%98%EB%B9%84-4034032/>


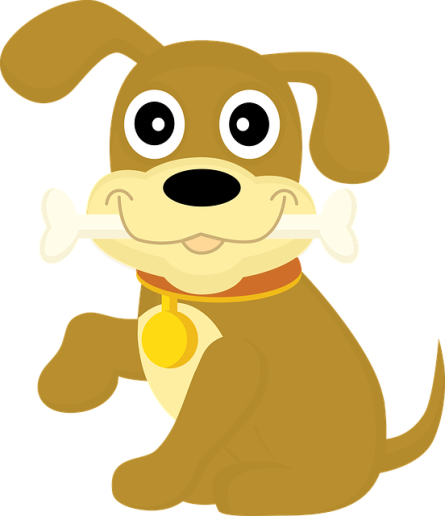


Supplementary figure 4. Picture prompt #2

<https://pixabay.com/ko/vectors/%ea%b0%9c-%eb%82%a8%ec%84%b1-%eb%bc%88-%ec%b9%bc%eb%9d%bc-%ec%83%88%eb%81%bc-1305702/>


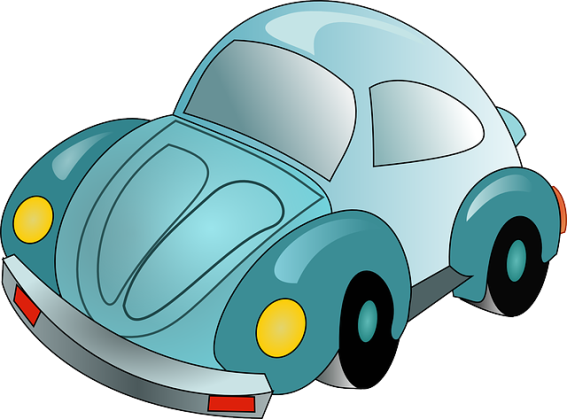


Supplementary figure 5. Picture prompt #3

<https://pixabay.com/ko/vectors/%ec%8a%b9%ec%9a%a9%ec%b0%a8-%ec%b0%a8-%ec%9e%90%eb%8f%99%ec%b0%a8-%eb%8a%99%ec%9d%80-155267/>


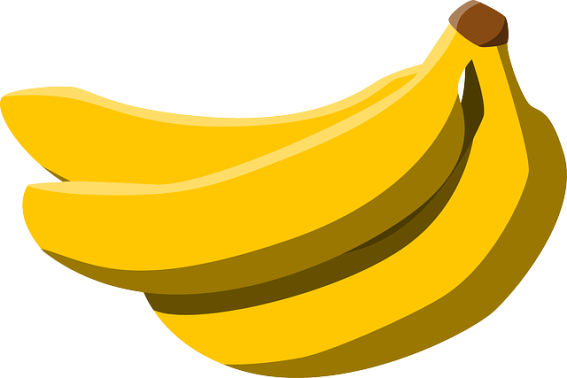


Supplementary figure 6. Picture prompt #4

<https://pixabay.com/ko/vectors/%eb%b0%94%eb%82%98%eb%82%98-%ec%9d%8c%ec%8b%9d-%ea%b3%bc%ec%9d%bc-%eb%85%b8%eb%9e%80%ec%83%89-146688/>

**Guideline for Eliciting Three Types of Joint Attention**

**Operational definitions of three types of joint attention:**

1. **Initiation of joint attention (IJA)**

The participant shifts their gaze from the toy to the examiner and back to the toy or from the examiner to the toy and back to the examiner. This behavior is elicited if observed within 10 seconds since presenting of toy object by the examiner.
Failure to show IJA is also a meaningful behavior; therefore, each toy object should be placed for a minimum of 10 seconds to fully capture such “absence of IJA”.

1. **Low level response to joint attention (RJA_low)_**

The participant shifts gaze by turning their head towards toy object (Toy 1) the examiner is pointing at. Meaningful gaze-shift is presumed to have occurred if maintained for at least a full 1 second. If the participant shifts gaze to look at Toy 2 (distractor), then RJA_low_ is not elicited.

1. **High level response to joint attention (RJA_high_)**

The participant shifts gaze by turning their head towards picture the examiner is pointing at. Meaningful gaze-shift is presumed to have occurred if maintained for at least a full 1 second. If the participant shifts gaze anywhere other than the picture examiner points to, then RJA_high_ is not elicited.


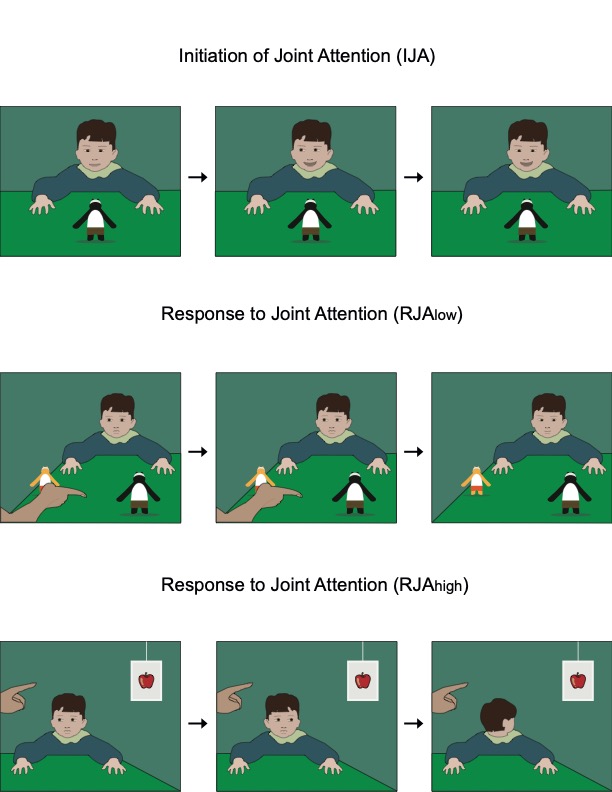


Supplementary figure 7. Joint attention types and operational definitions

**Specific guideline for eliciting each joint attention type:**

- All trials are conducted with both the examiner and participant seated at a table as shown in ‘Joint Attention Camera and Task Setup.’

*Initiation of joint attention*

A. Interaction situation using a single toy

(0) During initiation phase, the examiner is not allowed to speak or give any verbal instructions to the participant.

(1) Place one toy on the table at designated spot and observe the participant. Give the participant at least 10 seconds to initiate an interaction using the toy, by simply looking at the toy or making eye contact with examiner or shifting gaze from toy to examiner (or vice versa).

(2) If the participant makes eye contact with examiner, then examiner should respond by making eye contact with the participant. If the participant looks at the toy, then examiner may then look at the toy to mimic the participant’s behavior.

(3) In a particular order of the examiner’s choice, use each of the 5 toys, each time giving at least 10 seconds for the participant to start initiation of joint attention. Present the same toy twice in a row after 10 second pause between each presentation.

*Response to joint attention*

A. Interaction situation using two toys (one ‘stimulus toy’ and one ‘distractor toy’)

(1) Place two toys on the table, at designated spots. Toy 1 is placed adjacent to the camera, right across from the participant, and Toy 2 is placed on the far table corner, across from the examiner. While setting up, examiner must be careful not to block the camera view.

(2) Initiate by calling the participant’s name (name-calling is allowed up to three times). Then, making a pistol grip, point at Toy 1 with index finger for at least 5 seconds.

(3) Remove the first pair of toys from sight and then re-present them after 10 seconds. Then repeat the same process using the next four toy pairs.

B. Interactive situation using pictures mounted on either wall

(1) Initiate by calling the participant’s name (no more than three times). Then, turn to face Picture #1 (from the participant’s point of view, located on the right wall, bottom corner) and using index finger point at the picture for at least 5 seconds.

(2) After a short pause, repeat the previous step to elicit RJA using Picture #1 again.

(3) After a short pause, repeat the previous step to elicit RJA using Picture #1 for the third time.

(4) After a short pause, turn to face Picture #2 (from the participant’s point of view, located on the left wall, bottom corner) and using index finger point at the picture for at least 5 seconds.

(5) Repeat previous step two more times.

(6) Follow the same process but only repeat once using Picture #3 (from the participant’s point of view, located on the right wall, top corner) and Picture #4 (from the participant’s point of view, located on the left wall, top corner).

**Number of trials (repeated elicitation of joint attention behavior) per task type**

- IJA : 5 toys x 2 tries = 10 trials
- RJA_low_ : 5 pairs of toys (for 1 toy of interest, 1 distractor toy) x 2 tries = 10 trials
- RJA_high_ : Picture#1 x 3 tries + PIcture#2 x 3 tries + Picture#3 x 2 tries + Picture#4 x 2 tries = 10 trials

**Scoring Guideline for Three Types of Joint Attention**

- Upon completing joint attention tasks following the above guideline, complete the Yonsei Joint Attention Scoring Sheet to rate the participant’s joint attention performance.
- The Scoring Sheet is self-explanatory, requiring counting the number of times the participant showed ‘eye contact’ or ‘gaze shift’ during the entire course of joint attention trials.
- A true Initiation of Joint Attention (IJA) behavior is observed, by definition, when there is both eye contact with the examiner and shifting of gaze from toy to examiner (or vice versa).
- Response to joint attention is observed, by definition, when the participant turns their head to look at either toy object or picture object the examiner points to for at least a full one second while stimulus (examiner’s finger pointing) is presented.


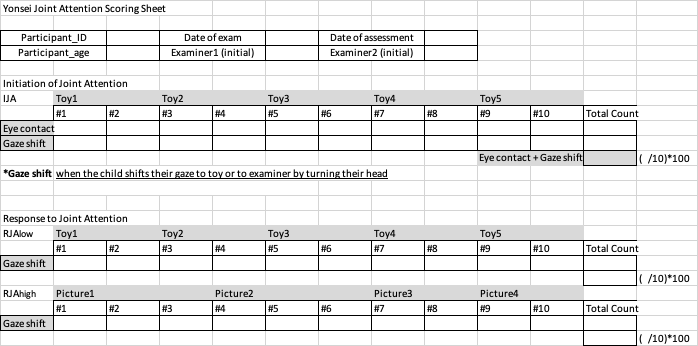


Supplementary table 1 Yonsei Joint Attention Scoring Sheet
