# Supplementary material for: Protocol for the development of joint attention-based subclassification of autism spectrum disorder and validation using multi-modal data
Source: BMC Psychiatry. 2023 Aug 15;23:589. doi: 10.1186/s12888-023-04978-4 (PMC10426216; doi:10.1186/s12888-023-04978-4)
Supplement: Supplementary file 2 — Supplementary Material 2. Social gaze-based clustering analysis standard operating procedure. [file 12888_2023_4978_MOESM2_ESM.docx]

**Social Gaze-Based Clustering Standard Operating Procedure**

Yonsei-Seoul Multi-modal Subclassification (YSMS)

**Contents:**

**Recording Joint Attention Situation for Eye Gaze Estimation**

- **Setting up the RGB-D camera**
- **General flow of recording**

**Acquired Data—Video**

**Joint Attention Tasks and Eye Gaze Patterns**

**Gaze pattern-based clustering analysis**

- **Calculating Head Pose Values for Gaze Pattern Imputation**
- **Semi-supervised Clustering Analysis Based on Social Gaze Patterns**

**Equipment—Recording Device**

**Recording Joint Attention Situation for Eye Gaze Estimation**

1. Connect RGB-D camera such as Azure Kinect device to your computer using the USB-C cable. Ensure that properly connected and recognized by the system.
2. Install the Azure Kinect SDK provided by Microsoft (or any equivalent for other RGB-D device). The SDK includes the necessary drivers, APIs, and tools to interact with the Azure Kinect device.
3. Configure the recording parameters: set up the desired recording parameters such as frame rate, resolution, and sensor options. Choose to record color camera data and depth data (i.e. 1080p HD, 30 frames per second)
4. Create a recording object: in your application or script, create a recording object using the Azure Kinect SDK. This object will handle the recording process and provide methods to start, stop, and manage the recording.
5. Start the recording: call the appropriate method in the recording object to start the recording. This will begin capturing data from the Azure Kinect device according to the specified parameters.
6. Record the desired data: Following the ‘Joint Attention Task Protocol’ (see separate document for details) record while conducting three types of joint attention. While the recording is in progress, the Azure Kinect device will continuously capture data from its sensors. The recorded data will include color frames and depth frames.
7. Stop the recording: once you have captured the desired data, call the appropriate method in the recording object to stop the recording. This will finalize the recording and save the captured data to a file on your computer.

**Acquired Data—Video**

- A joint attention videoclip is acquired from each participant while conducting joint attention tasks. Each video frame of a joint attention videoclip will capture the participant’s response to the three types of joint attention tasks. By default, participant is sitting in front of the camera, either engaged in the task by turning their head to see objects of interest located in the middle and either far lefthand side or righthand side of the front-facing view plane or distracted—looking at “distractor” toy object or areas other than where prop objects used in the joint attention experiment.

**Joint Attention Tasks and Eye Gaze Patterns**

- The joint attention task carefully designed in a manner such that the participant is required to turn their head at an angel of 45 degrees to the right or left (from the participant’s viewpoint) to observe objects of interest. The three elicited gaze patterns are looking down the middle or looking either left or right 45 degrees from imaginary midline.
- Participants with age-appropriate joint attention skills will show all three elicited gaze patterns while those with poor joint attention skills will show little or no elicited gaze patterns.
- The task order is as follows: initiation of joint attention (IJA) 🡪 low level response to joint attention (RJA_low_) 🡪 high level response to joint attention (RJA_high_)
- Unless participant is too upset to proceed through the full sequence of joint attention tasks, the videorecording for joint attention and social gaze analysis is to be done in one take, where initiation of joint attention and response to joint attention is elicited multiple times (multiple social gaze incidents).

Supplementary figure 8. Joint attention task—gaze pattern acquisition setup

**Calculating Head Pose Values for Gaze Pattern Imputation**

- A logical assumption since all the props used in the experiment fall on the same dimension or imaginary vertical hyperplane parallel to the participant’s head/eyes, head pose values such as yaw, pitch, and roll may be used to impute eye gaze patterns.
- Using C++ or python language-based computer vision (i.e. OpenCV) or face recognition libraries (Dlib), automated face detection per video frame and head pose values may be estimated for each face using state of the art face recognition models.
- We use a modified algorithm for head pose value estimation – utilizing Dlib Face Recognition model for detecting face with 68-point face landmarks, reconstruction of 2D face from 2D RGB images with depth information from depth images.


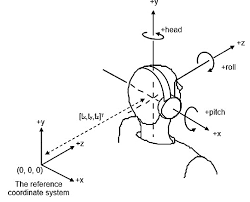


Supplementary figure 9. Head pose values—yaw, pitch, and roll

<https://www.pinterest.co.kr/pin/825566175434796782/>


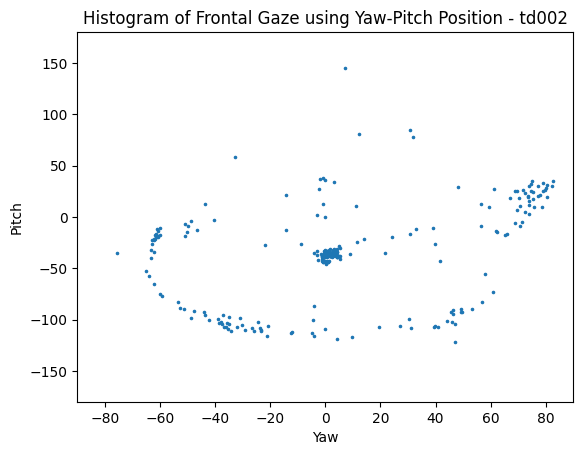


Supplementary figure 10. Example of yaw vs pitch graph (gaze pattern) of one participant

**Semi-supervised Clustering Analysis Based on Social Gaze Patterns**

Concept

- To label subtypes by joint attention gaze patterns, we compute summary statistics for each head-pose feature per participant.
- Summary values consist of mean, standard deviation, skewness, and kurtosis—which can be calculated using the ‘yaw’, ‘pitch’, ‘roll.’
- The multi-index columns are flattened then feature scaling is applied.


Supplementary table 2 Example summary values derived from yaw, pitch, and roll

- Use “good responder” and “poor responder” gaze patterns as labeled reference to use small amount of labeled data to help improve the performance of model trained with a large amount of unlabeled data.

Procedure

- Cluster all data without considering the “good responder” label. This will allow to visualize the natural structure or distribution of the data.
- After seeing the results, use “good responder” labeled data to label the clusters that most closely alight with these labels.
- Adjust clustering algorithm or parameters based on this analysis to better align the clusters with the “good responder” labels.
- End outcome is 2 or 3 subtypes based on social gaze patterns.


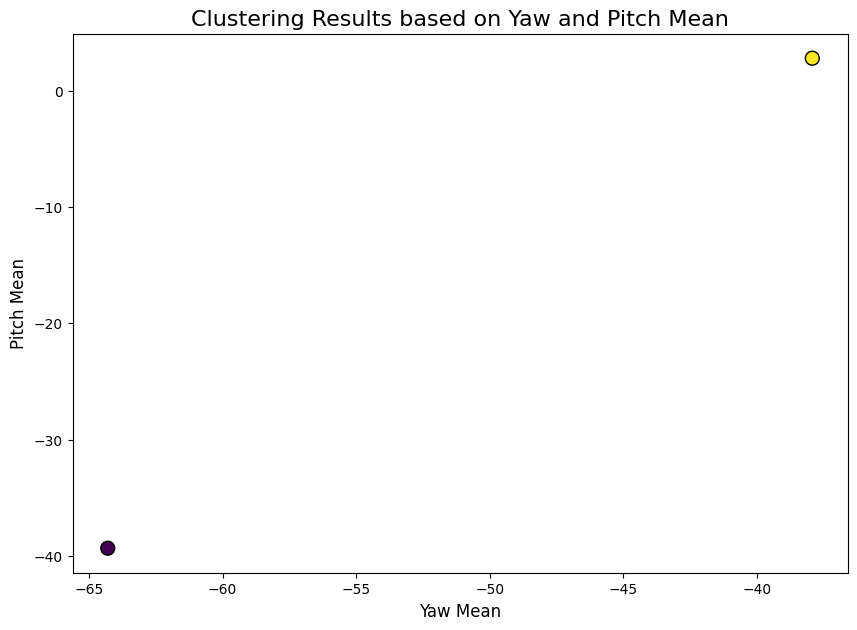


Supplementary figure 11. Example clustering results with two participants with different social gaze patterns


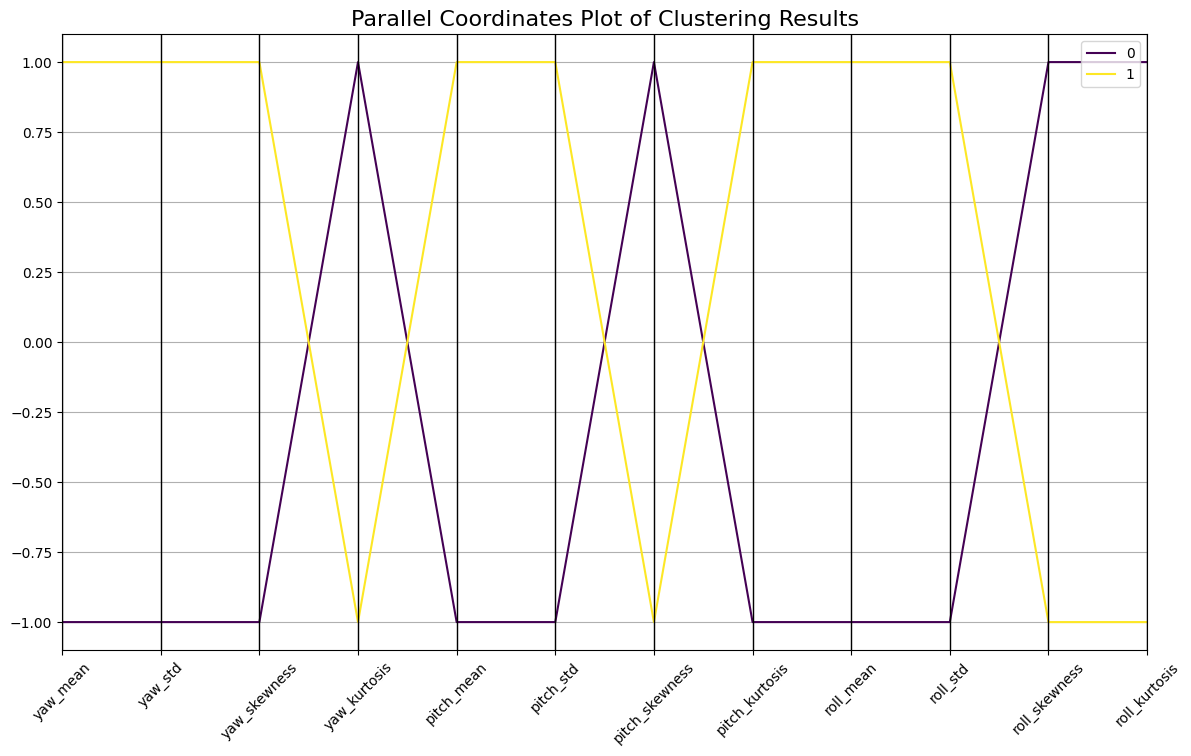


Supplementary figure 12. Example clustering results with summary statistics visualized for two participants with different social gaze patterns

**Equipment – Recording Device**

- Any RGB-D camera is acceptable for the purpose of conducting this study protocol; however, depending on the specific camera model, different camera parameter settings & calibration may be required.
- When using RGB-D camera, device must be connected to host PC and powered properly. System requirements (supported operating systems, hardware) may vary depending on the camera model.
- RGB camera could also be used as there are codes that can estimate head pose and gaze by 3D reconstruction of the face without the use of the depth information. However, more accurate estimation of head-pose and gaze may be possible with depth information that can be collected using an RGB-D camera’s depth sensor.
